# Environmental Pollution in Shanghai Hospital Departments in Terms of PM_2.5_

**Published:** 2019-04

**Authors:** Erbao GUO, Henggen SHEN

**Affiliations:** 1. College of Environmental Science and Engineering, Donghua University, Shanghai, China; 2. College of Environment and Energy Engineering, Anhui Jianzhu University, Hefei, China

## Dear Editor-in-Chief

“Hospital-acquired respiratory system infection is associated with hospital indoor aerosols, which functions as the carrier of virus diffusion by adhering to aerosol particles” (1–3). Different departments each face their own unique threats to air quality. The outpatient and inpatient departments suffer from crowds as well as from the accumulation of various pathogens. Surgical smoke, also known as aerosol, refers to suspensions of fine particles created by the cutting and ablation of tissues using high-frequency electrotomes, laser scalpels, or ultrasonic scalpels (4). Beyond just immediate symptoms of possible headache, blepharitis, and mucositis, long-term exposure to surgical smoke increases the frequency of cancer incidence (5).

A typical general hospital in Shanghai was selected as the object of this study. The PM_2.5_ concentrations in the different departments and their adjacent outdoor environment were monitored continuously from Mar 6, 2016 to Mar 16, 2016. The measured results revealed the PM_2.5_ concentrations in the outpatient and inpatient departments are influenced by the outdoor PM_2.5_ concentrations, the type of ventilation, and the crowd densities. The surgical department usually suffer from severe surgical smoke pollution. The PM_2.5_ number concentration peaked near the breathing zone of medical staff while they performed cutting or coagulation procedures. Interactions of thermal plumes caused by surgical smoke and laminar flows from the ceiling lead to the accumulation of fine particles in the vicinity of the surgery table.

The average PM_2.5_ concentrations after lunch break were significantly greater than that in the lunch break in the inpatient department and the outdoor environment (Table 1). As for the outpatient department, the higher average PM_2.5_ concentrations in the room were due to the lunch break, as, during lunch break, the air conditioning systems were switched off. The PM_2.5_ concentration in the outpatient department was almost constant after the lunch break when the air conditioner operated until 17:00. Regardless of time before and after lunch break, the average PM_2.5_ concentrations in the inpatient department office were greatest (>75 μg/m^3^) at four monitoring sites. After the lunch break, the average PM_2.5_ concentrations in the outpatient department were lowest at four monitoring sites, and the air there qualified as Class B air quality (GB3095-2012), whereas the average PM_2.5_ concentrations at the other three sites exceeded 75 μg/m^3^.

**Table 1:** Levels of PM_2.5_ at four sites during the test

***Sampling site***	***PM_2.5_ (μg/m^3^)***					
	***Lunch break (13:00–14:00)***			***After lunch break (14:00–17:00)***		
	***Mean±SD***	***Minimum***	***Maximum***	***Mean±SD***	***Minimum***	***Maximum***
Outdoor	74.8±1.7	72.0	80.0	84.2±7.3	70.0	96.0
Ward, inpatient department	55.2±5.7	46.0	68.0	82.8±15.5	47.0	122.0
Office, inpatient department	99.0±21.7	71.8	155.5	149.7±25.2	83.9	219.1
Outpatient department	57.0±4.8	49.8	65.5	47.6±2.7	43.8	58.6

PM_2.5_ concentrations in the inpatient department office were significantly greater than those in the other departments and the outdoor (all *P*<0.001). Meanwhile, PM_2.5_ concentrations in the ward and the outdoor area were significantly greater than those in the outpatient department (all *P*<0.001).

Moreover, our statistical results demonstrated that PM_2.5_ concentrations were significantly greater after the lunch break than that during the lunch break in the inpatient department and the outdoor (all *P*<0.001).

**Fig. 1:**
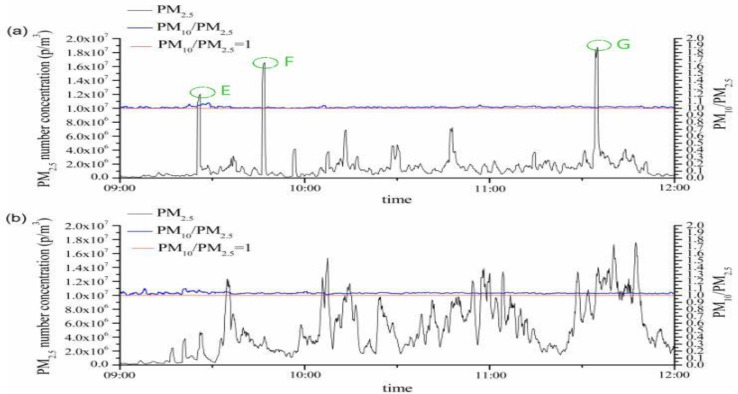
PM number concentration curves during the test: (a) at the astral lamp; (b) at the surgery table

Fine particles (PM_2.5_) account for most of PM_10_ in number concentration, but a small number of coarse particles (PM_10_-PM_2.5_) raised by the surgical smoke plumes are governing mass concentration during cutting or coagulation performed. The PM_2.5_ and PM_10_ number concentrations exhibited similar trends (Fig. 1). The maximum observed PM_2.5_ and PM_10_ number concentrations were 1.8781 × 10^7^ p/m^3^ and 1.8910 × 10^7^ p/m^3^ at Peak G, respectively. Furthermore, the actual particle concentration in the breathing zone could be even greater than the detected levels. Usually, the surgeon is at a working distance of 20–40 cm from the point of surgical smoke generation. Under the action of thermal buoyancy, the highest concentration of surgical smoke plume passes upward directly into the operating surgeon’s facial field, so the operating surgeon is always exposed to the highest concentrations of the plume, whereas other medical staff are exposed over a greater time period.

PM_2.5_ and PM_10_ number concentrations of surgical smoke were not significantly different at the astral lamp in the operating room (*P*=0.706). Meanwhile, PM_2.5_ and PM_10_ number concentrations of surgical smoke were not significantly different at the surgery table in the operating room (*P*=0.214). Moreover, our statistical results demonstrated that PM_10_ mass concentrations were significantly greater than PM_2.5_ mass concentrations at the astral lamp and the surgery table in the operating room (all *P*<0.001)
